# Visualization and Bibliometric Analysis of Carbon Neutrality Research for Global Health

**DOI:** 10.3389/fpubh.2022.896161

**Published:** 2022-07-06

**Authors:** Linghao Mao, Yiling Zhu, Chunhua Ju, Fuguang Bao, Chonghuan Xu

**Affiliations:** ^1^School of Management Engineering and E-Business, Zhejiang Gongshang University, Hangzhou, China; ^2^School of Foreign Languages, Zhejiang Gongshang University, Hangzhou, China; ^3^Contemporary Business and Trade Research Center, Zhejiang Gongshang University, Hangzhou, China; ^4^Academy of Zhejiang Culture Industry Innovation and Development, Zhejiang Gongshang University, Hangzhou, China; ^5^School of Business Administration, Zhejiang Gongshang University, Hangzhou, China

**Keywords:** carbon neutrality, knowledge map, research hotspots, CiteSpace, human health

## Abstract

The visual analysis of carbon neutrality research can help better understand the development of the research field and explore the difficulties and hot spots in the research, thus making contributions to “carbon emission reduction,” environmental protection and human health. This paper makes a visual quantitative analysis of 2,819 research papers published in top international journals from 2008 to 2021 in the WOS core database. It is found that China, the United States, Britain, and Germany are leading the way in carbon neutrality research. The research hotspots are mainly divided into three dimensions: (1) biomass energy and the negative effects it might bring; (2) ways and methods of electrochemical reduction of carbon dioxide; (3) catalysts and catalytic environment. The research mainly went through the conceptual period of 1997–2007, the exploration period of bioenergy from 2008 to 2021, the criticized period of bioenergy sources from 2011 to 2013, and the carbon dioxide electroreduction period from 2013 to the present. In the future, the research direction of biomass energy is to find one kind of biomass energy source which can be stored in a low-carbon way, produced in large quantities at a low cost, and will not occupy forestland. The electrolysis of water to produce hydrogen and the synthesis of fuel with CO_2_ are two major research directions at present, whose aims are to find the suitable catalyst and environment for the reaction. Besides, more research can be done on “carbon neutrality” policies so as to reduce carbon dioxide emissions from the source, develop a low-carbon economy and protect human health.

## Introduction

At present, more than 120 countries and regions have put forward the goal of carbon neutrality, which has become one of the common goals of the world. The concept of carbon neutrality was first introduced in 1997 by Future Forests (later renamed Carbon Neutral), a company based in London, UK. It means that households and individuals can offset their carbon emissions by purchasing certified carbon credits for environmental protection purposes. The company also provides carbon reduction services such as afforestation for these users. In 2006, the New Oxford English Dictionary named “carbon neutral” as its word of the year, and the term was then officially added to the dictionary. The dictionary defines it to protect the environment by calculating the amount of carbon dioxide emitted and then absorbing it by planting trees. Then “carbon neutral” began to appear in academic journals. At present, the research on carbon neutrality is mainly divided into four parts: the first is the research on biomass energy, from the initial crop production of biomass fuel to forest wood, and then to microalgae. Microalgae is one of the most suitable bioenergy energy sources at present. Finding suitable sources of biomass energy is the focus of research in this area (1–4); the second is the research on the reduction of carbon dioxide to hydrocarbon fuels, the main paths are carbon dioxide capture, water and carbon dioxide decomposition, and the synthesis of fuels. The current research focus in this field is to find suitable electrocatalysts to achieve high-efficiency synthesis of fuels (5–7); the third is the exploration of new energy sources, such as the use of electrolysis of water to generate hydrogen energy, and the search for suitable photoelectrode materials to convert solar energy (8).

The above research content is about the exploration of the realization path of carbon neutrality, which has certain reference value for the relevant research. But these studies fail to give an overall picture of carbon neutrality research. At present, there are few literature reviews in the field of carbon neutrality, and none of them can directly show the development, research hotspots and future trends of carbon neutrality research. In general, there is a lack of literature on the overall development context and research hotspots in the field of carbon neutrality.

Therefore, this paper uses Citespace and Alluvial Generator as visual measurement soft-wares to carry out the following research from the dimensions of “countries,” “institutions,” “co-citation analysis of literature,” and “co-occurrence of keywords:” (1) research status and cooperative relations in the field of carbon neutrality among various countries; (2) research hotspots and emerging trends in the field of carbon neutrality; (3) the development of carbon neutrality research and the difficulties encountered in the research.

The main contributions of this paper are summarized as follows:

1. A bibliometrics research framework is designed to provide research methods and visualization approaches for research hotspots, research development, and research trends in different fields.
2. The international situation of carbon neutrality research is visualized and cooperation among different countries in this field are analyzed to further promote international co-operation in carbon neutrality research.
3. The visual analysis of research hotspots, research development and research trends of carbon neutrality research is carried out to identify the development trend and some urgent problems in the research, so as to provide suggestions and implications for the future development of carbon neutrality research.

In Section 2, the experimental design of this paper is proposed, including the realization method and the manifestation of the visualization of carbon neutralization research. In Section 3, the hotspots and development of carbon neutralization research are visually analyzed. In Section 4, we present our research conclusions, limitations, and put forward suggestions for the development of carbon neutrality research. Finally, we give our conclusions and future research directions in Section 5.

## Research Design

### Research Methods

**Figure 1:**
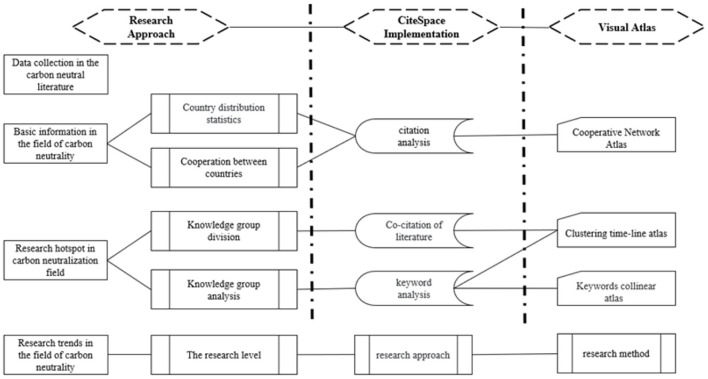
The analysis diagram of carbon neutral development.

For the quantitative analysis of carbon neutrality research, this paper uses the visualized literature analysis tool CiteSpace developed by Professor Chen Chaomei (9). The literature information on the theme of carbon neutrality is used to draw the scientific knowledge map through citation analysis, co-citation analysis and co-occurrence of keywords. The map is interpreted by the clustering timeline, time zone and co-occurrence map with the help of word frequency, intermediary centrality, and emergent analysis. The development trend of this field can be summarized by exploring the evolution stages, changes of research topics, research hotspots and research frontiers of carbon neutrality research. The analysis of the development of carbon neutrality research combined with CiteSpace technology is shown in Figure 1.

### Data Collection

On 13 February 2022, the author selected articles and reviews on the theme of “carbon neutral” and “carbon neutrality” through WoS Core Collection database for data retrieval of unlimited years. Given the reliability of the conclusion, 2,819 valid data were selected by excluding articles with incomplete abstracts and key information or inconsistent themes. Among them, there are 2,392 articles and 427 reviews. Then the titles containing citation information are exported, and the exported data are de-duplicated by CiteSpace 5.8. R3.

## Research Results and Analysis

### Visual Analysis of Basic Information on Carbon Neutralization Research

In order to explore countries worthy of attention and find leaders to promote carbon neutrality research, this paper uses the cooperation analysis of CiteSpace software to study the influence and cooperation relationships among countries, providing a theoretical basis for the current situation and contribution analysis of carbon neutrality.

**Figure 2:**
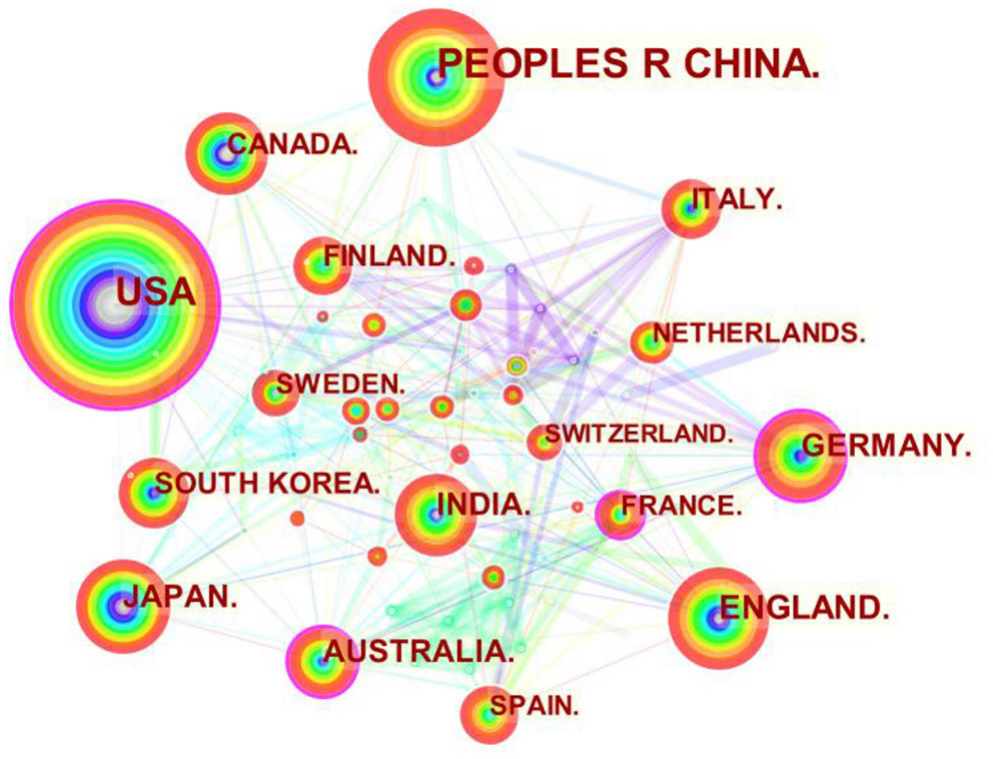
The knowledge map of major partnerships in high-yielding countries conducting carbon neutrality studies.

CiteSpace is used to carry out visual statistical analysis of 2,819 top journal articles, and the knowledge map of the cooperative relations between high-yielding countries conducting carbon neutrality research is obtained after being adjusted with visualization, as shown in Figure 2. Figure 2 shows a total of 102 nodes and 369 links. The connection strength of links is calculated using cosine distance.

**Figure 3:**
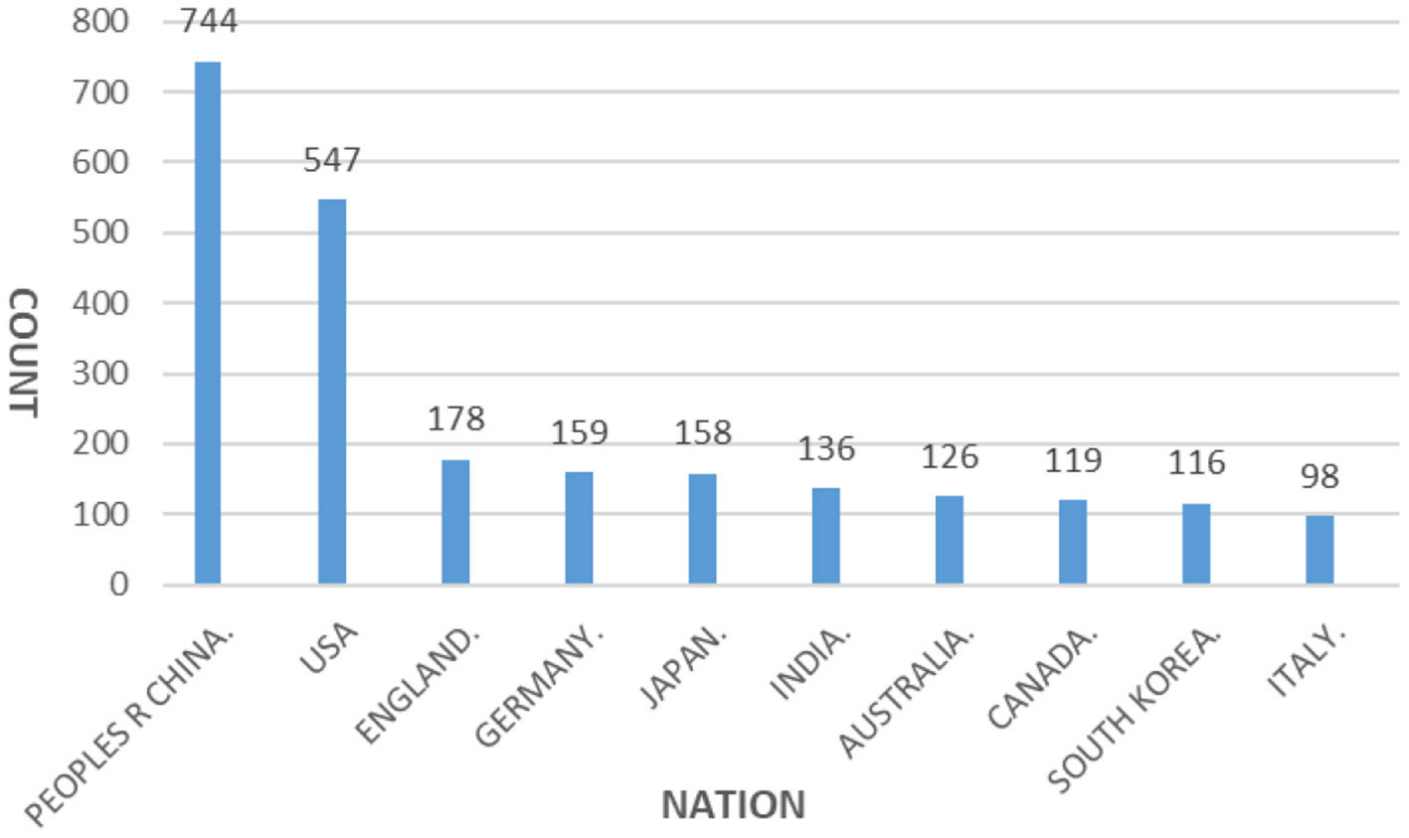
Distribution of the number of published articles in high-yielding countries conducting carbon neutrality studies.

**Figure 4:**
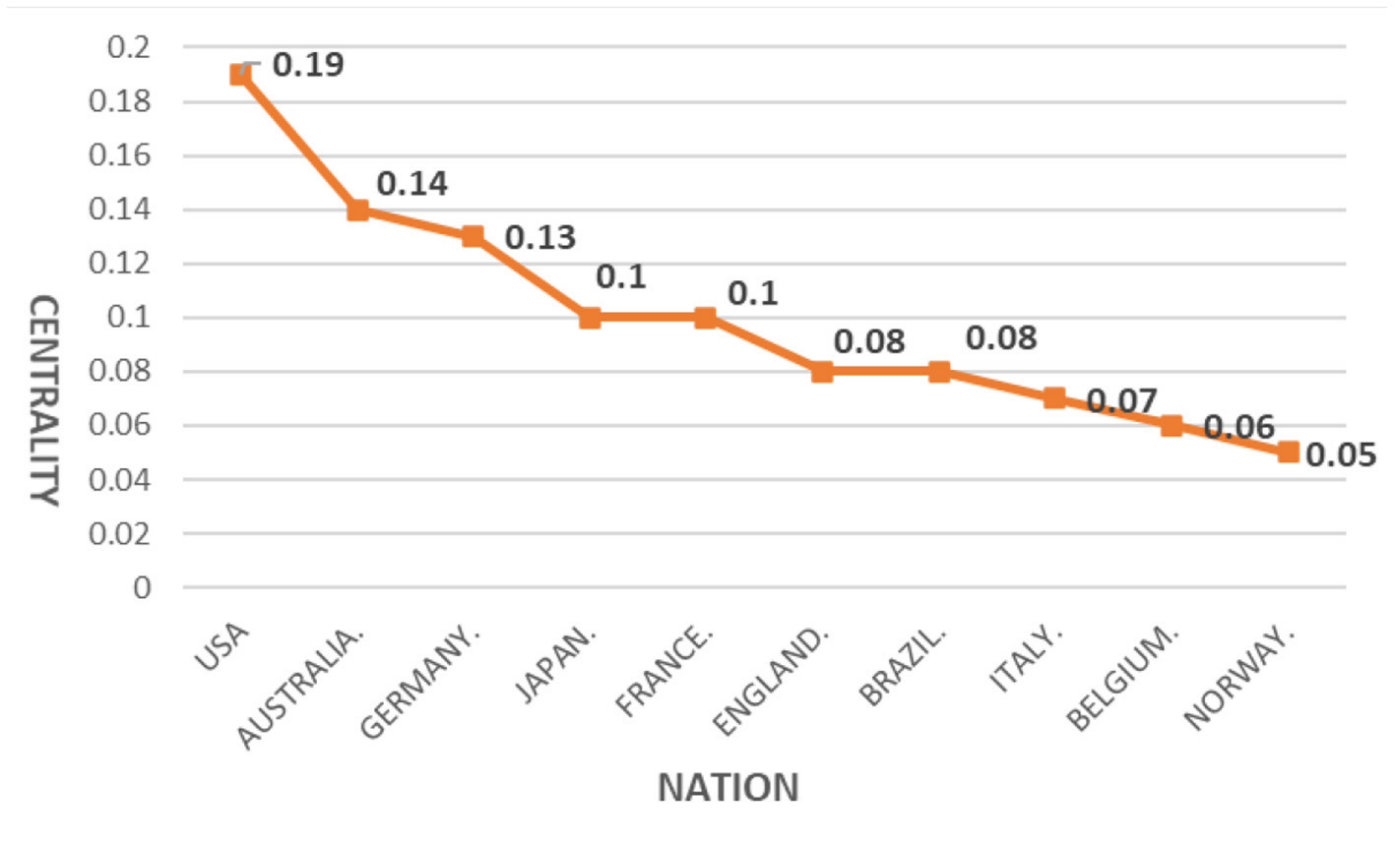
Distribution of countries with high centrality in the study of carbon neutrality.

In Figure 2, a node represents a country, the size of the node represents the number of publications, the width of rings in different colors represents the number of publications in different years, and the years change from far to near as the ring rings change from inside to outside. The link between the nodes represents the cooperative relationship between the two countries, and the width of the link represents the closeness of the cooperative relationship. After sorting out and analyzing the information in Figures 2–4 are obtained, which are used to further analyze the relationship between high-yielding countries in the study of carbon neutrality. The following conclusions are drawn from the study.

From the perspective of cooperative relations, there is no strong direct cooperative relationship between high-yielding countries in the study of carbon neutrality. For example, there is less cooperation between high-producing countries such as China, the United States, the United Kingdom, Germany, and Japan. On the one hand, it is because that high-yielding countries have more domestic resources can complete research in their own countries. On the other hand, high-yielding countries such as China and the United States have a lot of technological competitions, and a lot of studies are confidential. Low-yield countries, however, cooperate more with other countries. One is to work with low-yielding countries and integrate their resources for further study; the other is to work with high-yielding countries, relying on their resources.

In terms of the number of papers published, China, the United States, the United Kingdom, Germany, and Japan are the leading countries in carbon neutrality research. The paper output of China and the United States is far ahead of that of the United Kingdom, Germany and Japan. Among these countries, the United States has a long history of research than China, and it has a relatively stable number of publications since 2008. While in China, the number of papers published has surged after the government proposed a carbon-neutral target in 2020.

From the perspective of centrality, nodes with the centrality <0.1 are taken as key nodes, which are also considered as innovative nodes and leaders of reform and development in this field. The United States, Australia, Germany, Japan, and France are all key nodes in the study of carbon neutrality. Among these nodes, the centrality of China with the largest number of publications is only 0.03. The reason is that there is less cooperation between China and other countries, and most of the research results are completed in China. Also, China' s publications are mainly concentrated after 2020. Therefore, for better development, China should strengthen international cooperation in carbon neutrality research, to be more innovative and competitive.

### Visual Analysis of Carbon Neutrality Research Hotspots

#### Analysis of Research Hotspots Based on Literature Co-citation

**Figure 5:**
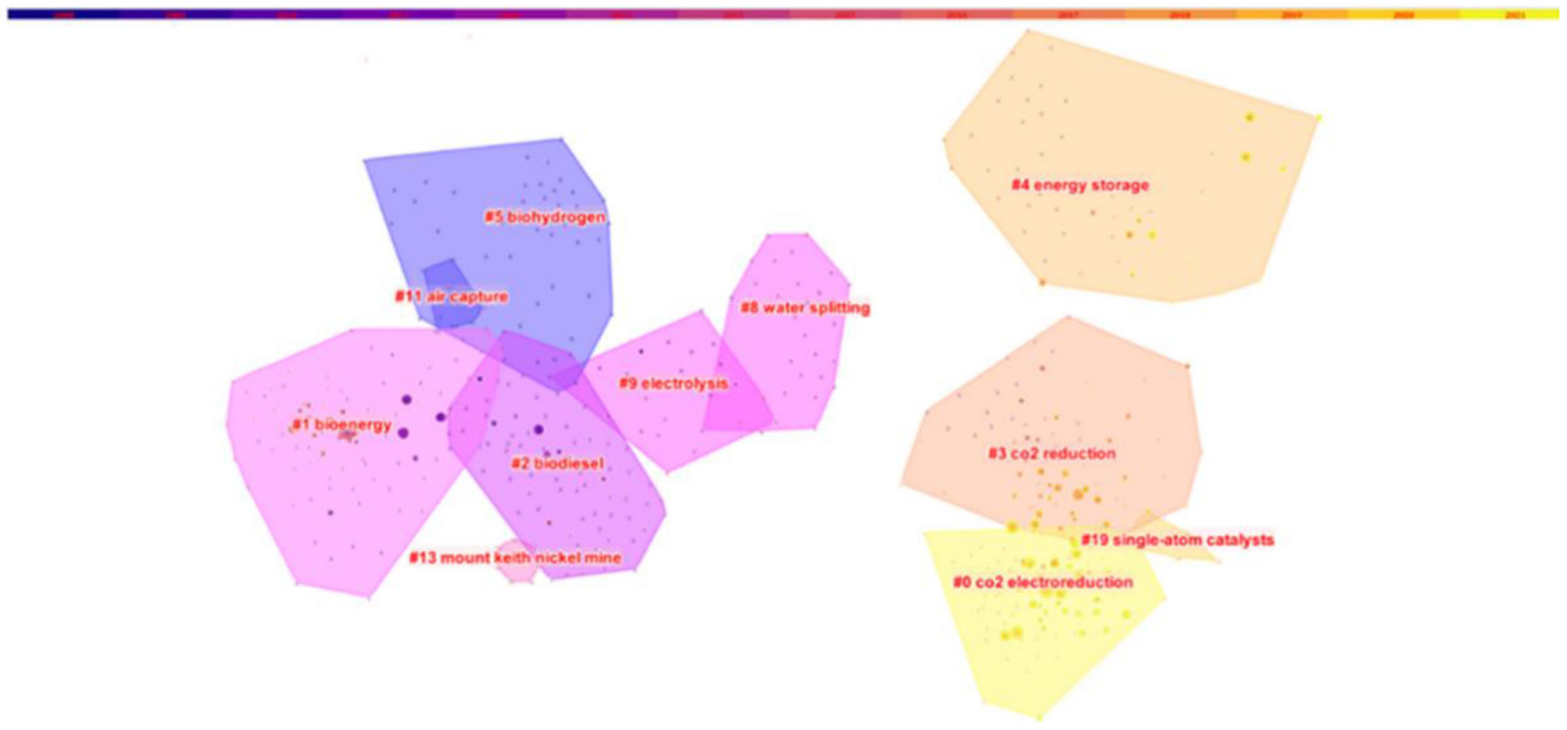
The co-citation map of literatures on carbon neutrality.

In 1973, American information scientist Small first proposed the concept of co-citation as a research method to measure the degree of relationship between documents. At the same time, Marshakova, a Soviet intelligence scientist, also proposed this concept as Small did. The co-citation of literature means that two papers (or more papers) are simultaneously cited by one or more subsequent papers. Then these two papers are said to constitute a co-citation relationship, and this kind of relationship will change with time. The development and evolution of a discipline can be explored through the study of co-citation network of literature. By studying the literature on carbon neutrality, the co-citation map of the literature on carbon neutrality is obtained, which contains 789 nodes and 2,929 links. The co-citation map of the literature is shown in Figure 5.

Figure 5 shows the panoramic map of carbon neutrality research. In order to better observe the change of the theme, nodes and links have been hidden. Areas with different colors show the main clustering distribution of carbon neutrality research, and the sequence number before the theme label increases in ascending order. The color of the area gradually becomes shallower from left to right, and the time also gets closer from left to right. This map reveals the knowledge clustering of carbon neutrality research and the spatio-temporal evolution of hot topics. Extract the information in Figure 5 and cluster the sorted data. Clustering is shown in Table 1 based on different algorithms.

**Table 1:** Clustering based on different algorithms.

**Number**	**Size**	**Label (LLR)**	**Label (MI)**	**Mean (year)**
0	94	CO_2_ electroreduction	Geometric modulation	2017
1	91	Bioenergy	Global warming potential	2010
2	80	Biodiesel	Microalgal biomass	2008
3	69	CO_2_ reduction	3d electrodes	2013
4	51	Energy storage	Techno-economic analysis	2014
5	43	Biohydrogen	Triacyl glycerides	2006
8	30	Water splitting	Photosystem ii	2010
9	22	Electrolysis	Lime kiln	2008
11	9	Air capture	Air capture	2006
13	8	Mount keith nickel mine	Mount keith nickel mine	2011
19	5	Single-atom catalysts	Single-atom catalysts	2017

Table 1 shows clustering based on LLR algorithm and MI algorithm. The calculation logic of LLR algorithm in literature is:

There are two keywords: keyword i and keyword j. K11 represents the number of literatures containing both keyword i and keyword j, K12 represents the number of literatures containing keyword i but not keyword j, K21 represents the number of literatures containing keyword i but not keyword j, and K22 represents the number of literatures containing neither keyword i nor keyword j. N is the total number of references, S is the similarity value, Hm is the matrix entropy, Hr is the information entropy obtained by adding each row, and Hc is the information entropy obtained by adding each column. The calculation of S is shown in formula (1)–(4).


(1) $$\begin{array}{lll} S & = & 2\times \left(Hm-Hr-Hc\right) \end{array}$$

(2) $$\begin{array}{l} Hm=-(\frac{K11}{N}\log(\frac{K11}{N})+\frac{K12}{N}\log(\frac{K12}{N})+\frac{K21}{N}\log(\frac{K21}{N})\\ \;+\frac{K22}{N}\log(\frac{K22}{N})) \end{array}$$

(3) $$\begin{array}{l} Hr=-(\frac{K11+K12}{N}\log(\frac{K11+K12}{N})\\ \;+\frac{K21+K22}{N}\log(\frac{K21+K22}{N})) \end{array}$$

(4) $$\begin{array}{l} Hc=-(\frac{K11+K21}{N}\log(\frac{K11+K21}{N})\\ \;+\frac{K21+K22}{N}\log(\frac{K21+K22}{N})) \end{array}$$

Based on the actual research on carbon neutrality, the clustering obtained by LLR algorithm is more consistent with the actual situation of carbon neutrality research, and MI algorithm has a certain reference value. In the table, “(CO_2_ electroreduction, geometric modulation), (bioenergy, global warming potential), (biodiesel, microalgal biomass), and (CO_2_ reduction, 3d electrodes)” are the largest clusters. The average year refers to the average publication year of literatures in the cluster. The later the year, the more advanced the cluster is. As can be seen, the initial research on carbon neutrality began in 2006 with the theme of “air capture,” the absorption of carbon dioxide from the air through chemical reactions (10). By 2008, themes were “electrolysis,” “biodiesel” and “microalgal biomass,” aiming at reducing carbon dioxide emissions by using clean energy such as biomass produced by microalgae and hydrogen produced by water electrolysis instead of traditional carbon-containing energy sources (11, 12). By 2010, the research topic was still around the biological energy and hydrogen energy proposed in 2008, and the source of biomass energy and its possible harm were also discussed. After 2013, the theme of “CO_2_ reduction” and “CO_2_ electroreduction” mainly focused on catalytic production of energy from carbon dioxide, and catalysts contained liquid catalysts, silver catalysts, and so on (13).

#### Evolution Path Analysis of Research Hotspots Based on Emergent Literature

The text analysis of key node literature is helpful to explore the key path and knowledge inflection point of carbon neutrality research evolution. The dynamic characteristics of a topic can be reflected by the abnormal increase of the number of literature citations. The sudden literature represents the research hotspot of a certain event, and the centralized analysis of the sudden literature can study the evolution path in this field. In this paper, the information of Figure 5 is extracted and sorted out for sudden document detection and the information is shown in Table 2. Among them, “begin” and “end” represent the start and end time of the emergence of hot spots, and the emergent intensity reflects the degree of attention of hot literature.

**Table 2:** Top 16 references with the strongest citation bursts (2008–2021).

**Number**	**References**	**Strength**	**Begin**	**End**
#1	Chisti Y, 2007, BIOTECHNOL ADV, V25, P294	13.68	2008	2012
#2	Searchinger T, 2008, SCIENCE, V319, P1238	11.18	2009	2012
#3	Fargione J, 2008, SCIENCE, V319, P1235	10.72	2009	2013
#4	Searchinger TD, 2009, SCIENCE, V326, P527	9.6	2010	2013
#5	Hu Q, 2008, PLANT J, V54, P621	6.97	2010	2012
#6	Mckechnie J, 2011, ENVIRON SCI TECHNOL, V45, P789	12.65	2011	2016
#7	Cherubini F, 2011, GCB BIOENERGY, V3, P413	10.99	2012	2016
#8	Repo A, 2011, GCB BIOENERGY, V3, P107	7.32	2012	2015
#9	Holtsmark B, 2012, CLIMATIC CHANGE, V112, P415	6.25	2013	2016
#10	Qiao JL, 2014, CHEM SOC REV, V43, P631	7.41	2017	2019
#11	Lu Q, 2014, NAT COMMUN, V5, P0	6.95	2017	2019
#12	Zhang S, 2014, J AM CHEM SOC, V136, P1734	5.55	2017	2019
#13	Kortlever R, 2015, J PHYS CHEM LETT, V6, P4073	5.94	2018	2021
#14	Gao S, 2016, NATURE, V529, P68	5.44	2018	2021

The publication time of #1–#5 was between 2007 and 2009, and the emergence time was concentrated between 2008 and 2012. #1 and #5 explored the utilization of biomass energy, and proposed that microalgae, rich in oil, can be used as an alternative and renewable source of biofuel raw materials, and is a potential renewable and carbon-neutral alternative to petroleum fuels. The way of economic competition between microalgae biodiesel and petroleum diesel was discussed, and the expression of genes related to fatty acid synthesis in microalgae was explored (14, 15). #2–#4 discussed the possible harm caused by unreasonable use of biomass energy. #2 found that farmers around the world transformed forests and grasslands into new farmlands to plant biofuel raw materials for profit, which would reduce the absorption of greenhouse gases. The model found that ethanol using corn as raw material did not save 20 % of carbon dioxide emissions, but nearly doubled the greenhouse gas emissions in 30 years (16). #3 mentioned that converting tropical rain forests, peatlands, tropical grasslands or grasslands to produce biofuel based on food crops would create a “biofuel carbon debt” that exacerbates greenhouse gas emissions. Only biofuels produced from biowaste do not generate “biofuel carbon debt” (17). #4 mentioned that the current assessment of carbon emissions of biomass energy was flawed, and the emission changes caused by land use during harvesting or planting of biomass energy for energy were not calculated (18).

The publication time of #6–#9 was concentrated from 2011 to 2013, and the emergence time was concentrated from 2011 to 2016. The main content of the study was as follows: Due to the extensive criticism of food crops biofuels, people's attention turned to the second generation of wood biofuels. The literature studied whether wood obtained from vast forests should be used as a source of wood-based biofuels and whether this would be an effective climate policy. The study found that logging was not a carbon neutral activity. Forest-based bioenergy would increase total carbon emissions, and obtain biomass energy from residues was beneficial, but producing ethanol from standing trees would increase total greenhouse gas emissions for 100 years. A method for estimating the impact of CO_2_ emissions from biomass combustion on climate was proposed, and a method for calculating the indirect emissions from the production of bioenergy by using logging residues was introduced (19–22).

#10–#12 were published between 2012 and 2014, and the emergence time was concentrated between 2017 and 2019. The research hotspot was to convert carbon dioxide into fuel by using catalysts. #10 reviewed the research progress of electrocatalysts used for the reduction of carbon dioxide (CO_2_) to produce low-carbon fuels in recent years, such as the influencing factors of the performance of electrocatalysts and the challenges faced by electrocatalysts, and put forward several research directions for practical application, aiming at mitigating performance degradation, overcoming additional challenges, and promoting the development of the research in this field (23). #11 reported a nano porous silver electrocatalyst that could reduce carbon dioxide to carbon monoxide with about 92 % selectivity at a rate (i.e., current) more than 3,000 times higher than that of polycrystalline catalyst (24). #12 explored the preparation of SnO_2_ nanocrystals with high specific surface area by hydrothermal method, and considered it as an excellent electrocatalyst for CO_2_ reduction to formic acid (25).

#13–#14 were respectively published in 2015 and 2016, and its emergence time has been from 2018 to now, which is a hot topic in current research. #13 reviewed several aspects of electrochemical reduction of carbon dioxide, such as introducing several heterogeneous and molecular electrocatalysts for CO_2_ reduction, and discussing the reaction paths of their formation of various products (26). #14 explored the conversion of CO_2_ into CO to replace fossil raw materials, preparing two kinds of four atomic layers, namely pure cobalt metal layer, cobalt metal and cobalt oxide coexistence layer, to activate CO_2_ (27).

#### Research Hotspots and Evolution Analysis of Carbon Neutrality Based on Keyword Emergence

Keywords are selected from the title, abstract and text of the paper, which are words of substantial significance to express the central content of the paper. The emergent analysis of keywords can find out the keywords frequently used by scholars in a certain period and their development trends, and can also judge the degree of activity and emerging trends of a field. This paper selects the literature on personalized research from 2008 to 2021 for keyword prominence analysis. Table 3 shows the 19 strongest emergent words of carbon neutrality research from 2,819 high-level literature.

**Table 3:** Top 19 keywords with the strongest citation bursts.

**Keywords**	**Strength**	**2008–2021**	**Begin–End**
Biofuel	14.61	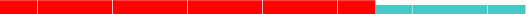	2008–2017
Energy	6.18	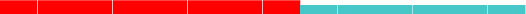	2008–2015
Fuel	5.84	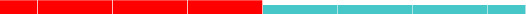	2008–2014
Biodiesel	5.62	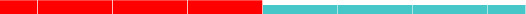	2008–2014
Carbon sequestration	7.3	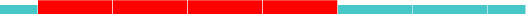	2009–2016
Vegetable oil	5.66	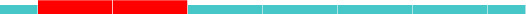	2009–2012
Ethanol	5.34	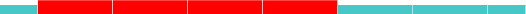	2009–2016
Bioenergy	8.3	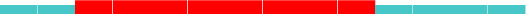	2010–2017
Escherichia coli	6.62	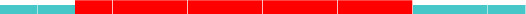	2010–2018
Climate change	9.26	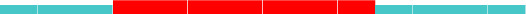	2011–2017
Management	5.31	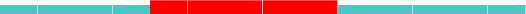	2012–2016
Balance	5.12	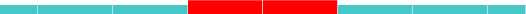	2013–2016
Pyrolysis	4.92	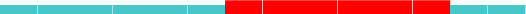	2014–2019
Dioxide	5.34	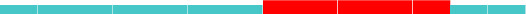	2015–2019
Generation	4.82	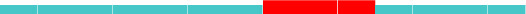	2015–2017
CO_2_ reduction	6.82	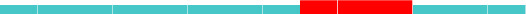	2016–2018
Hydrogen	5.21	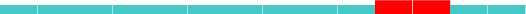	2018–2019
Bio oil	4.69	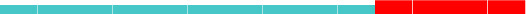	2018–2021
Electroreduction	10.93	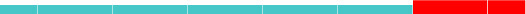	2019–2021

It can be seen from Table 3 that the earliest emerging keywords, the keywords with the longest duration, and the keyword with the highest intensity is “Biofuel,” and the latest keyword is “electroreduction.” The duration of research hotspots on carbon neutrality shows a trend from long to short. Key words such as “biofuel, energy, fuel, biodiesel, carbon sequestration” emerged from 2008 to 2011, and these research hotspots lasted for 6 years on average. From 2012 to 2019, keywords such as “balance, pyrolysis, dioxide, generation, CO_2_ reduction, and hydrogen” were highlighted, and the average duration of research hotspots was 2 to 3 years. The above highlighted words reflect the research hotspots and evolution trends of carbon neutrality in different periods. The emergence of the keywords “bio oil, electroreduction” has continued to this day, and the research involved in these two keywords are hot topics of carbon neutrality research in the future. Based on the analysis of the key words and the above literature emergent research, it can be found that the initial research on carbon neutrality is bioenergy, while “biofuel” and “biodiesel” are the research hotspots and “vegetable oil” is the evolution of hotspots. Because bioenergy will bring a certain degree of “biofuel carbon debt,” crop-based bioenergy and forest wood-based bioenergy have been proved to be likely to aggravate the greenhouse effect. After 2014, the research focus shifted from bioenergy to “electroreduction” and CO_2_ synthetic energy was replaced by fossil raw materials through the study of electrocatalysts.

#### Research Hotspots of Carbon Neutrality Based on Collinear Keywords

The keyword co-occurrence map is conducive to analyzing the research hotspots in a field. The more frequent a keyword appears, the more popular the field is. Through the analysis of 2,819 articles in the field of carbon neutrality, a visualization chart of the co-occurrence of keywords in carbon neutrality research is obtained, as shown in **Figure 8**. After pruning, there are 390 nodes and 1,221 connections in the graph. The size of nodes represents the frequency of keywords, and nodes with frequency >50 in the figure show labels.

Keywords co-occurrence graph analysis shows that keywords with the highest frequency are energy, carbon dioxide, impact, performance, climate change, and carbon neutrality. Combined with the above key literature research, the research hotspots obtained from the co-occurrence analysis of the above keywords can be summarized into three categories: the first is the research on biomass energy, such as the exploration of raw material sources of biomass energy, the future of biomass energy and so on; the second is the study of the possible negative impacts of biomass energy, such as the carbon emission calculation model of biomass energy; the third is the ways and methods of electrochemical reduction of carbon dioxide, including the research on catalyst and catalytic environment (28–30).

### Visual Analysis of Carbon Neutrality Research Trends

#### Research Trend Analysis of Carbon Neutrality Research Based on Keyword Frontier Time Zone Diagram

**Figure 6:**
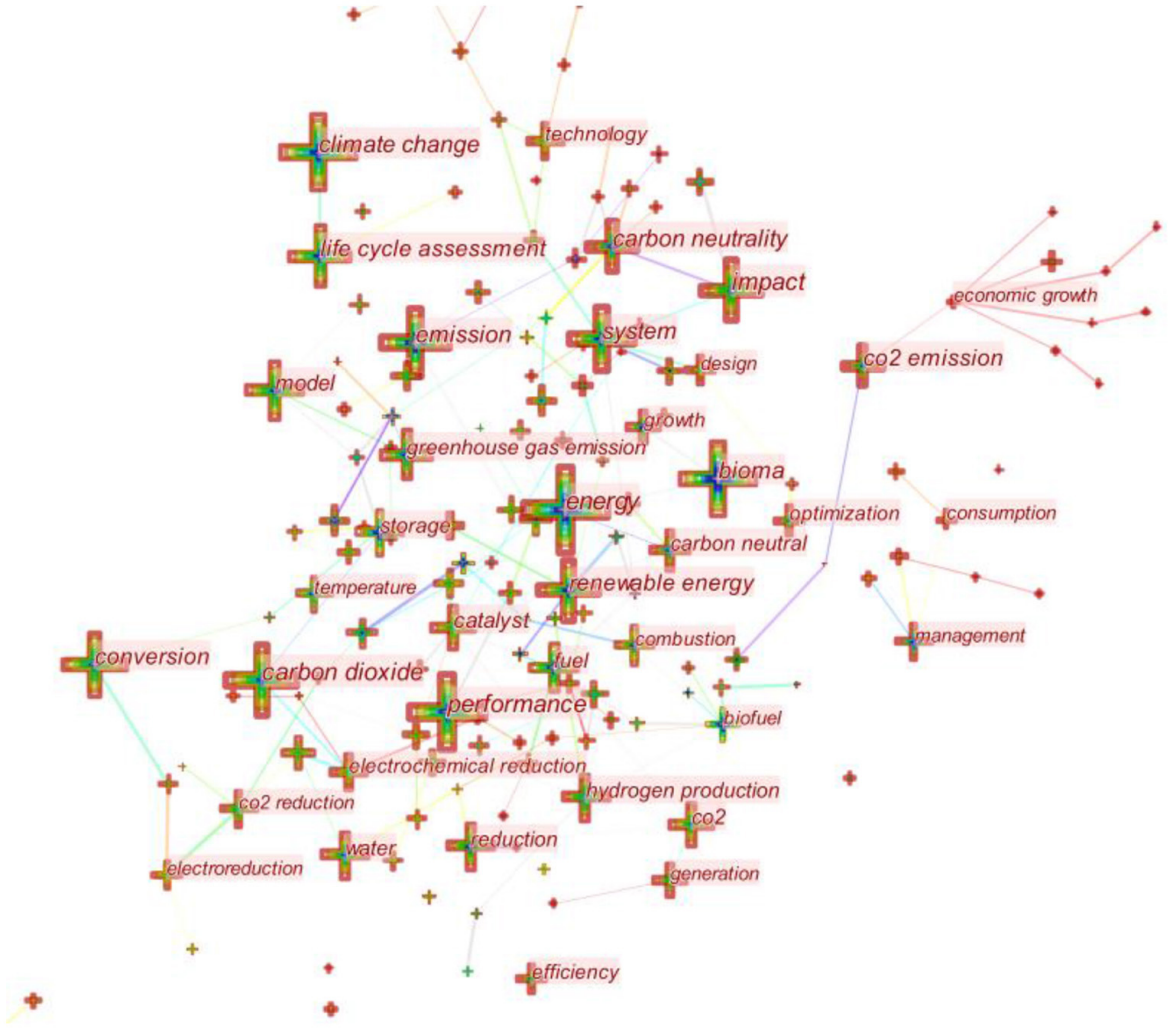
Keywords co-occurrence map.

**Figure 7:**
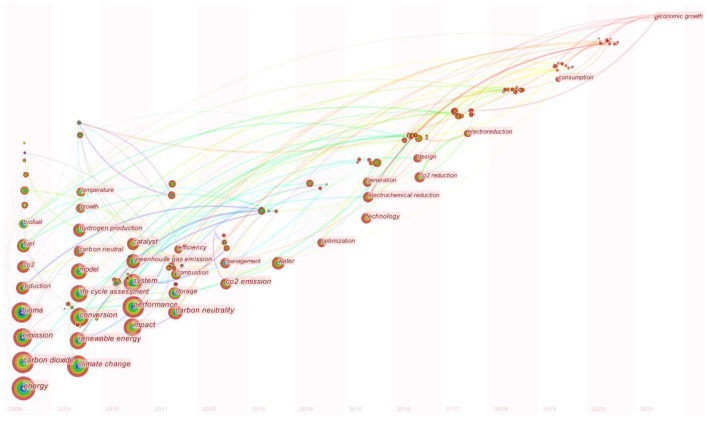
The frontier time zone diagram for keywords.

The frontier zone view of keywords is generated according to the interaction of hot topics. Each column represents a year, and the location of the node represents the main distribution time of the keyword, that is, the hot time of the topic. Figure 7 is a time zone diagram of the keyword frontier generated according to keyword collinearity in Figure 6. According to Figure 7 and the above analysis, the development of carbon neutrality can be divided into four stages.

Phase I: The concept period is from 1997 to 2006. The concept of carbon neutrality was put forward, carbon emissions, bio-energy and clean energy received attention, and the concept of carbon neutrality gained people's attention and support, and the proper term of carbon neutrality emerged.

Phase II: The bioenergy exploration period is from 2008 to 2011. During this period, people focused on bioenergy to realize carbon neutrality. “Life cycle assessment, renewable energy, and bioma” are all research hotspots in this period, and people are committed to finding a suitable biota as a raw material for bioenergy.

Phase III: The critical period of bioenergy sources is from 2011 to 2013. During this period, questions and criticisms were raised on the realization of carbon neutrality of bioenergy. The main topics are as follows: the first is to research on the sources of bioenergy, and it is found that although crops and forest wood as the sources of bioenergy reduce the emission of carbon dioxide, but also reduce the original absorption of carbon dioxide; the second is to study the carbon emission calculation model of biomass energy. Considering the indicators, such as plant carbon storage and future carbon dioxide absorption capacity, and discuss the practical role of biomass energy in carbon neutrality at present.

Phase IV: The carbon dioxide electroreduction period is from 2013 to the present. Electroreduction of carbon dioxide has become the main research focus, which is mainly divided into two directions: the first is to explore excellent electrocatalytic reductants; the second is to explore the best reaction environment for electroreduction of carbon dioxide. And in this period, hydrogen energy produced by electrolytic water has also attracted attention. Carbon neutrality is gradually linked to economic growth. How to achieve carbon neutrality while maintaining rapid economic growth may be one of the research hotspots in the future.

#### Trend Analysis of Carbon Neutrality Research Based on Impact Flow Diagrams

**Figure 8:**
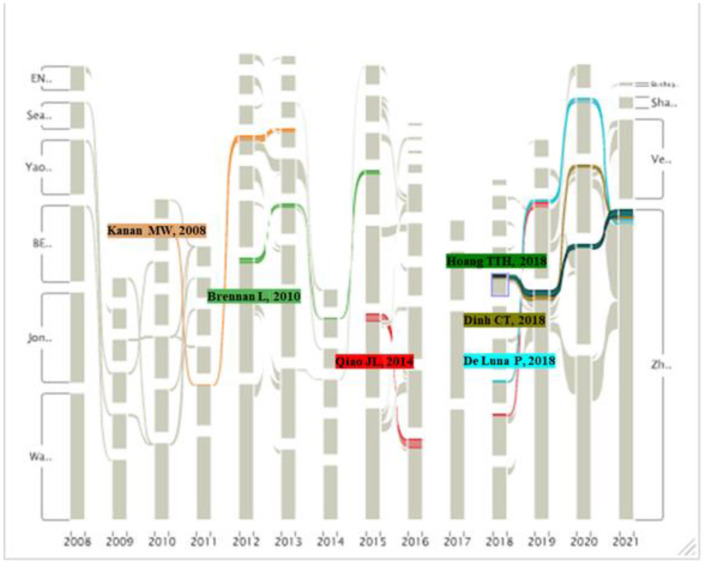
Impact flow diagram of highly cited literature.

This paper first uses CiteSpace to process data by selecting references with top 100 citation times from 2008 to 2021, and imports 14 network information files into the Alluvial Generator for visual analysis and data analysis. After module selection and module layout, this paper obtains the alluvial flow map of representative articles in the field of carbon neutrality as shown in Figure 8. The figure highlights six representative literatures which are cited consistently. It's found that Kanan (31) and Brennan (1) received continuous attention in the early time. The three highlighted literatures in 2018 are still attracting much attention, which may be viewed as one research direction in the future. It is worth noting that the highly cited literatures in 2017 are cited less in other year, and there may exist a turning point in the research direction of this year, which can be further studied.

Kanan (31) received continuous attention from 2010 to 2013. The literature studied a catalyst for “water splitting,” which could not only form in situ from earth-abundant materials but also operate in neutral water under ambient conditions (31). Besides, Brennan (1) also received much attention from 2012 to 2015. The literature criticized the first- and second-generation biofuels, and reviewed the technologies underpinning microalgae-to-biofuels systems, focusing on the biomass production, harvesting, conversion technologies, and the extraction of useful co-products (1). The literature of Qiao (23) was widely cited from 2015 to 2019. The literature studied the influencing factors of CO2 reduction electrocatalysts, analyzed the challenges in achieving highly active and stable CO2 reduction electrocatalysts, and proposed several research directions for practical application (23).

Literatures of Hoang (34), Dinh (32), and De (33), published in 2018, soon received continuous attention which continues to this day. They represent the research trend in the field of carbon neutrality, focusing on the CO_2_ electroreduction. They have, respectively studied the CO_2_ electroreduction to ethylene, the synthesis of highly efficient electrocatalysts for CO_2_ reduction and the improvement of selective electrocatalysts for CO_2_ reduction (32–34).

The analysis of the above representative literatures shows that:

1. The research on the hot spots and future trends of carbon neutrality in this paper is consistent with the research results of representative literatures, which can verify the rationality of the keyword analysis and emergent literature analysis in this paper.
2. The research focus of carbon neutrality has shifted from the preparation of biofuels to the CO_2_ electrocatalytic reduction, which is mainly divided into the reaction environment of electrocatalytic reduction, the synthesis of catalysts, and the improvement of catalysts. This is the current research hotspot as well as one of the main directions of future research.
3. Early ideas of carbon neutrality research were to reduce CO_2_ emissions, like using solar energy to produce clean energy or replacing fossil fuels with synthetic biofuels. However, due to the demand of social production and the difficulty of popularization, the current idea has changed into reducing the generated carbon dioxide and utilizing it as raw material to synthesize energy.

## Discussion

In this paper, CiteSpace software is used to make a visual analysis of 2,819 relevant literatures in carbon neutrality research from 2008 to 2021 by combining qualitative and quantitative analysis, and the following conclusions are drawn:

From the perspective of the research area, it is found that the leading countries in carbon neutrality research are China, the United States, Britain and Germany. From the analysis of high-yielding countries, high-yielding institutions and authors with high yield, China is far ahead, but China's foreign cooperation is too little and most of the literature was published after 2020. Therefore, in the field of carbon neutrality, the countries with high influence are still the United States, Australia, Germany, and other countries.

From the perspective of research innovation (35–38), countries with strong innovation in carbon neutrality research are the United States, Australia, Germany, Japan, France, and other developed countries, and one institution with strong innovation is Univ Toronto (Canada). It is found that these countries and institutions lead the development of global carbon neutrality.

From the perspective of research content (39–41), the research hotspots of carbon neutrality are “CO_2_ electroreduction, microalgal biomass, air capture, water splitting.” It is mainly divided into three directions: the first is to study biomass energy, such as exploring the source of raw materials and the future of biomass energy; the second is to study the possible negative effects of biomass energy, such as the carbon emission calculation model of biomass energy; the third is to study the ways and methods of CO_2_ electrochemical reduction, including the research of catalyst and catalytic environment.

From the perspective of research progress, the research mainly went through the concept period of 1997–2007 (a period when the concept of carbon neutrality was proposed), the bioenergy exploration period from *2008 to 2011* (a period when people focused on bioenergy to realize carbon neutrality), the critical period of bioenergy sources from *2011 to 2013* (a period when questions and criticisms were raised on the realization of carbon neutrality of bioenergy), and the CO_2_ electroreduction period from *2013 to the present* (a period when CO_2_ electroreduction has become the main research focus).

From the above research analyses, the following suggestions can be summarized:

Firstly, the research hotspots of the realization path of carbon neutrality range from biomass energy to CO_2_ electroreduction. At present, electroreduction catalyst is a hot spot. But the criticism of the use of biomass does not mean that biomass does not contribute to carbon neutrality, but that there is currently a lack of a suitable source of biomass. The source of this biomass energy requires that it can be obtained at low cost and on a large scale, and it cannot have too much carbon storage itself, and it does not occupy the existing forest land area during cultivation. Finding the suitable source of biomass energy would be a huge boost to achieving carbon neutrality.

Secondly, the current research focus of carbon neutrality is carbon capture, which achieves carbon neutrality by reducing carbon dioxide in the air. At present, the main research focuses on the exploration of high-quality electroreduction catalysts, hoping to reduce carbon dioxide into fuel to replace part of fossil raw materials. However, the research on new energy sources has not made obvious progress since 2008. The research on electrolysis of water to generate hydrogen energy proposed in 2008 can be used to explore new electrocatalysts like the electroreduction of carbon dioxide.

Thirdly, a research hotspot for carbon neutrality is economic growth after 2021. As there are many developing countries in the world, economic development should be considered when exploring paths of carbon neutrality. The realization path of carbon neutrality should consider the economic development of all countries in the world before it can be promoted on a large scale. Since carbon emission reduction is a global goal, future research, and decision-making should fully consider the differences in global economic development and shift to a low-carbon economy without affecting economic development. Only in this way can we effectively improve the global environment and ensure human health (42).

Finally, excessive greenhouse gas emissions will surely cause harm to human health. However, based on the current development of carbon emission reduction technology, the total emission of carbon dioxide will not decrease in the next two decades. Therefore, for the sake of human health, in addition to technically reducing the generation of greenhouse gases, people need to pay attention to the impact of policies on greenhouse gas emissions. When technological development hits a plateau, people can promote the development of policies to establish a punishment mechanism for excessive carbon emissions, so as to develop a low-carbon economy and reduce greenhouse gas emissions at the source.

This paper designs a research framework of bibliometrics to provide research methods and visualization approaches for research hotspots, research development and research trends in different fields. Other scholars can learn from this framework when doing visualization of literature research. It's hoped that the framework can be further improved and supplemented by them.

The limitation of this paper lies in the selection of data sets. In order to improve the quality of data sets, this paper only selects articles and reviews in the WOS core database, while some important research may be neglected. The literature research in this paper uses Citespace and Alluvial Generator to supplement and verify each other, but it fails to make a comparative argument with the literature review written in the traditional way, which may omit some important conclusions.

## Conclusions

This paper is the first one to visually analyze the research status, hotspots and development of carbon neutrality from the perspective of bibliometrics, and propose a research framework. The study has found that the leading countries in carbon neutrality research are China, the United States, the United Kingdom, and Germany. USA, Australia, Germany, Japan, and France are leaders of the reform and development in this field. The research hotspots are mainly divided into three domains: (1) biomass energy and the negative effects it might bring; (2) ways and methods of electrochemical reduction of carbon dioxide; (3) catalysts and catalytic environment. The research mainly went through the conceptual period of 1997–2007, the exploration period of bioenergy from 2008 to 2021, the criticized period of bioenergy sources from 2011 to 2013, and the carbon dioxide electroreduction period from 2013 to the present. In the future, the research direction of biomass energy is to find one kind of biomass energy source which can be stored in a low-carbon way, produced in large quantities at a low cost, and will not occupy forestland. The reaction environment, catalyst synthesis and catalyst improvement of CO_2_ electrocatalytic reduction are the current and even future research directions. In the future work, we will improve the principle of data filtering and select excellent articles from other databases. In addition, conclusions generated under this research framework are compared with the traditional literature review to analyze the advantages and disadvantages of our framework and further improve our theoretical framework.

## Data Availability Statement

The original contributions presented in the study are included in the article/supplementary material, further inquiries can be directed to the corresponding authors.

## Author Contributions

FB and CJ designed the study and conceived the manuscript. LM, YZ, and FB designed the study and conceived the manuscript. LM and YZ implemented the simulation experiments. FB and CX drafted the manuscript and were involved in revising the manuscript. All authors contributed to the article and approved the submitted version.

## Funding

This research is funded by Natural Science Foundation of Zhejiang Province (Nos. LQ20G010002 and LY20G010001), and the Soft Science Research Program of Zhejiang Province (No. 2021C25010). This research is supported by the project of China (Hangzhou) cross-border electricity business school (No. 2021KXYJ06), the Philosophy and Social Science Foundation of Zhejiang Province (No. 21NDJC083YB). Contemporary Business and Trade Research Center of Zhejiang Gongshang University (Nos. XT202103 and XT202105).

## Conflict of Interest

The authors declare that the research was conducted in the absence of any commercial or financial relationships that could be construed as a potential conflict of interest.

## Publisher's Note

All claims expressed in this article are solely those of the authors and do not necessarily represent those of their affiliated organizations, or those of the publisher, the editors and the reviewers. Any product that may be evaluated in this article, or claim that may be made by its manufacturer, is not guaranteed or endorsed by the publisher.
